# Intratracheally Inhalable Nifedipine-Loaded Chitosan-PLGA Nanocomposites as a Promising Nanoplatform for Lung Targeting: Snowballed Protection *via* Regulation of TGF-β/β-Catenin Pathway in Bleomycin-Induced Pulmonary Fibrosis

**DOI:** 10.3390/ph14121225

**Published:** 2021-11-26

**Authors:** Mohammed H. Elkomy, Rasha A. Khallaf, Mohamed O. Mahmoud, Raghda R. S. Hussein, Asmaa M. El-Kalaawy, Abdel-Razik H. Abdel-Razik, Heba M. Aboud

**Affiliations:** 1 Department of Pharmaceutics, College of Pharmacy, Jouf University, Sakaka 72388, Saudi Arabia; 2 Department of Pharmaceutics and Industrial Pharmacy, Faculty of Pharmacy, Beni-Suef University, Beni-Suef 62514, Egypt; rasha.mahmoud@pharm.bsu.edu.eg (R.A.K.); heba.aboud@pharm.bsu.edu.eg (H.M.A.); 3 Department of Biochemistry, Faculty of Pharmacy, Beni-Suef University, Beni-Suef 62514, Egypt; mohamed.omar@pharm.bsu.edu.eg; 4 Department of Clinical Pharmacy, Faculty of Pharmacy, Beni-Suef University, Beni-Suef 62514, Egypt; Raghda.hussien@pharm.bsu.edu.eg; 5 Department of Clinical Pharmacy, Faculty of Pharmacy, Modern University for Technology and Information, Cairo 12055, Egypt; 6 Department of Pharmacology, Faculty of Medicine, Beni-Suef University, Beni-Suef 62511, Egypt; asmaa.hussein@med.bsu.edu.eg; 7 Department of Histology, Faculty of Veterinary Medicine, Beni-Suef University, Beni-Suef 62511, Egypt; abdelrazak.osman@vet.bsu.edu.eg

**Keywords:** nifedipine, pulmonary fibrosis, intratracheal inhalation, chitosan-PLGA nanocomposites, bleomycin, TGF-β/β-catenin pathway

## Abstract

Pulmonary fibrosis is a serious ailment that may progress to lung remodeling and demolition, where the key participants in its incidence are fibroblasts responding to growth factors and cellular calcium swinging. Calcium channel blockers, like nifedipine (NFD), may represent auspicious agents in pulmonary fibrosis treatment. Unfortunately, NFD bears complicated pharmacodynamics and a diminished systemic bioavailability. Thus, the current study aimed to develop a novel, non-invasive nanoplatform for NFD for direct/effective pulmonary targeting *via* intratracheal instillation. A modified solvent emulsification–evaporation method was adopted for the fabrication of NFD-nanocomposites, integrating poly(D,L-lactide-co-glycolide) (PLGA), chitosan (CTS), and polyvinyl alcohol, and optimized for different physiochemical properties according to the 3^2^ full factorial design. Additionally, the aerodynamic behavior of the nanocomposites was scrutinized through cascade impaction. Moreover, the pharmacokinetic investigations were conducted in rats. Furthermore, the optimum formulation was tested in bleomycin-induced pulmonary fibrosis in rats, wherein fibrotic and oxidative stress parameters were measured. The optimum nanocomposites disclosed a nanosized spherical morphology (226.46 nm), a high entrapment efficiency (61.81%) and a sustained release profile over 24 h (50.4%). As well, it displayed a boosted in vitro lung deposition performance with a mass median aerodynamic diameter of 1.12 µm. Pharmacokinetic studies manifested snowballed bioavailability of the optimal nanocomposites by 3.68- and 2.36-fold compared to both the oral and intratracheal suspensions, respectively. The intratracheal nanocomposites revealed a significant reduction in lung fibrotic and oxidative stress markers notably analogous to normal control besides repairing abnormality in TGF-β/β-catenin pathway. Our results conferred a compelling proof-of-principle that NFD-CTS-PLGA nanocomposites can function as a promising nanoparadigm for pulmonary fibrosis management.

## 1. Introduction

Pulmonary fibrosis is one of the most serious ailments that provokes vast mortality around the world. There are three types of pulmonary fibrosis, according to histological classification: typical interstitial pneumonia, fibrotic nonspecific interstitial pneumonia, and airway-centered fibrosis [1]. Pulmonary fibrosis may be triggered by diverse insults, like exposure to poisonous agents or drugs, incongruous autoimmune response, infection, or lung trauma [2]. Though some drugs as N-acetylcysteine, corticosteroids, and cytotoxic agents have been exploited for lung fibrosis management, they exhibited less noteworthy progression [3]. Till now, no specific lung-directed treatment for pulmonary fibrosis has been evolved. Therefore, searching for novel lung targeting drug modalities, markedly amend the progressive causal mechanisms, for management of pulmonary fibrosis is mandatory.

The key players in the emergence of pulmonary fibrosis are fibroblasts, in which various changes are aroused with subsequent damaging inflammatory responses in lung alveoli. Fibroblasts respond to various growth factors and cytokines as transforming growth factor beta (TGF-β), which exerts its impact through regulation of kinase pathways as phosphoinositide 3-kinase and mitogen-activated protein kinase. Additionally, it was claimed that TGF-β exerts its influence in tissue fibrosis *via* elevation of intracellular calcium levels, an action mediated by increments in the entry of extracellular calcium, alongside the release of intracellular sequestered calcium [4]. There are numerous types of calcium channels; among them are voltage-sensitive calcium channels (L and T types), which regulate the entry of external calcium. Calcium channel blockers which are selective to L and T type channels have been clinically harnessed for tackling of hypertension and regulation of heart rate [5]. Our hypothesis was to employ the L-type calcium channel blocker, nifedipine (NFD), for tackling pulmonary fibrosis *via* interfering with the calcium influx to fibroblasts through suppressing the effect of TGF-β1.

NFD, a dihydropyridine calcium antagonist, acts primarily on the calcium channels allocated on all cell surfaces, wherein its chief sites of action are the heart and smooth muscle cells. It is highly efficacious in the management of aortic regurgitation, hypertension, and vasospastic angina, as well as chronic angina rather than unstable angina [6]. Despite its broad range of clinical and therapeutic impacts, NFD bears complex oral pharmacodynamics and an aberrant bioavailability pursuant to its sparse aqueous solubility, besides a dual enzymatic metabolism mediated *via* P450 reductases, as well as CYP3A, resided into the gastric and small intestinal tissues [7]. Administered orally, NFD concentrations which are warranted to accomplish fruitful influences might be accompanied by considerable adverse effects, encompassing gastritis and amplified deaths for coronary heart ailed patients. Moreover, other side effects, such as diarrhea, notable hypotension, liver toxicity, and cerebral contusion might manifest with current NFD oral preparations [8]. Accordingly, the aforementioned encumbrances could be consistently mitigated through an alternate administration route for NFD. Hence, NFD lung targeting might be deemed as an auspicious strategy for the local treatment of pulmonary fibrosis, meanwhile abrogating most of its deleterious oral side effects.

Pulmonary targeting enables systemic drug delivery through the airway surfaces to the blood circulation. Embedding a drug into an adequately tailored, inhalable carrier can optimize the inhaled drug therapeutic effects, guard it from the defense mechanisms of the lung, and boost both cellular and extracellular drug transport [9]. Indeed, drug administration by conventional inhalation methods encounters diverse obstacles, such as hampered deep drug penetration, due to branched respiratory tree configuration, alveolar macrophages uptake, mucociliary clearance, poor aqueous drug solubility in the biological medium, in addition to the existence of thick viscous mucous, leading to drug absorption hindrance with a subsequent demand for high drug dosing [10]. Thus, great attention has been grabbed to polymeric nanoparticles to surmount the challenges of local drug administration for the management of lung diseases.

Intriguingly, the exploitation of biodegradable polymeric nanoparticles represents a promising maneuver for medical nanotechnology and smart dosage forms [11,12]. Poly(D,L-lactide-co-glycolide) (PLGA) is a biocompatible and biodegradable polymer with versatile degradative kinetics which is particularly hydrolyzed in vivo to yield resorbable monomers; glycolic and lactic acids, ensuring negligible systemic toxicity [13]. PLGA copolymer is accepted by the EMA, as well as the FDA, and has been thoroughly implemented for prolonged delivery of hydrophilic and hydrophobic drugs. Nevertheless, owing to its diminutive in vivo residence time on the mucosal surfaces, the absorption of PLGA-based drugs is robustly impeded. Surface functionalization with mucoadhesive polymers would be a salutary approach for extending the residence time of PLGA vectors onto the mucosal linings [14]. Chitosan (CTS) is immensely elected as a conjugating substance for PLGA nano-cargo, due to its mucoadhesivity, biocompatibility, biodegradability as well as penetration-enhancing characteristics over mucosal surfaces. Presumably, CTS mucoadhesiveness is inaugurated by the interaction among its cationic amino moieties with anionic domains, such as sulfonic and sialic acids of the mucosal membrane [15]. Therefore, in the present work, CTS surface decoration was pursued to consolidate the engagement of hydrophobic PLGA nanoparticles with the lung epithelia.

Assimilation of NFD into PLGA nanoparticles has been previously explored for different purposes [16,17]. Lee et al. [16] assessed the potential of intravenously-administered, NFD-laden PLGA nanoparticles for augmenting non-alcoholic fatty liver syndrome. Additionally, Zhao et al. [17] declared in vivo periodontal regeneration with topical application of NFD-based PLGA microspheres. However, the influence of processing variables on the physicochemical characteristics of PLGA nanoparticles, based on an experimental statistical design, was not investigated in these studies. Actually, an immaculate design approach for nano-cargo systematic optimization donates numerous merits, such as precision, accuracy, prognosis, as well as minimal experimental variability [18]. To the best of our knowledge, prior art did not disclose any clinical trial for inhalable NFD-CTS-PLGA nanocomposites for pulmonary fibrosis management.

Consequently, the aim of the current study was to explore the aptitude of CTS-PLGA nanoparadigm to accentuate the pulmonary targeting of NFD, thereby, precluding superfluous side effects concomitant with the oral route. To accomplish this goal, various variables affecting the characteristics of CTS-PLGA nanocomposites were scrutinized, employing a statistical full factorial design for marking the most desirable formulation and assessing their adequacy for pulmonary delivery. Additionally, cascade impactor was utilized for evaluating the aerodynamic characteristics of the assembled nanocomposites. Furthermore, in vivo pharmacokinetic studies were executed in rats for comparing the pharmacokinetic behavior of NFD following both oral and intratracheal inhalation. Moreover, the underlying molecular mechanism for the lung protective influence of NFD on bleomycin-induced pulmonary fibrosis in a rat model was inspected.

## 2. Materials and Methods

### 2.1. Materials

Nifedipine was kindly donated by Eipico (Cairo, Egypt). Poly(D,L-lactide-co-glycolide) (50:50; molecular weight 85,000 Da), polyvinyl alcohol (molecular weight 12,000–13,000 Da and 87–89% degree of hydrolysis), chitosan (low molecular weight 150,000 Da and 75–85% degree of acetylation), atorvastatin, sodium carboxymethyl cellulose, Tween 80 (polyoxyethylene sorbitan monooleate), ammonium acetate, acetonitrile (HPLC grade) and ethyl acetate (HPLC grade) were procured from Sigma-Aldrich (St. Louis, MO, USA). Dialysis bags with a molecular weight cut off of 12,000 Da were purchased from SERVA Electrophoresis GmbH (Heidelberg, Germany). Bleomycin injection was obtained from Cipla (Mumbai, India). All other enrolled ingredients were of analytical grade.

### 2.2. Preparation of NFD-CTS-PLGA Nanocomposites

A modified single emulsion–solvent evaporation technique, which is a superlative method for encapsulating lipophilic drugs, was adopted for the fabrication of NFD-CTS-PLGA nanocomposites [19]. In brief, precisely weighed quantities of PLGA and NFD (10 mg) were dissolved in acetone and dichloromethane, at a ratio of 1:1 *v/v,* which constituted the organic phase. Then, the organic phase (2.5 mL) was emulsified with 10 mL of the aqueous phase comprising PVA and 0.25% *w/v* chitosan which was prepared in 0.5% *v/v* acetic acid with a pH of 5 under homogenization (Ultra Turrax^®^ T 25 basic homogenizer, IKA, Staufen, Germany) at 13,500 rpm for 10 min. The ratio between the organic/aqueous phases was maintained at 1:4. For the organic solvents’ evaporation, the assembled nanocomposites dispersions were stirred on a magnetic stirrer at 600 rpm overnight at ambient temperature. The final nanodispersions were then kept at 4 °C in a refrigerator for subsequent investigations.

### 2.3. Statistical Design

To explore the influences of the various formulation moderators on the features of CTS-PLGA nanocomposites, the full 3^2^ factorial design within the Design-Expert^®^ software (version 12.0.3.0, Stat-Ease Inc. Minneapolis, MN, USA) was employed. Two causal factors were assessed in this design: *X*_1_: PLGA concentration and *X*_2_: PVA concentration, with two levels each. The entrapment efficiency percent of NFD (*EE%*: *Y*_1_), size of the nanocomposites (*Y*_2_), and accumulative % NFD released from the nanocomposites over 24 h (*Q*_24 *h*_: *Y*_3_) were elected as the dependent variables as outlined in Table 1. All probable combinations for tailoring NFD-CTS-PLGA nanocomposites are depicted in Table 2.

### 2.4. In Vitro Characterization of the Assembled NFD-CTS-PLGA Nanocomposites

#### 2.4.1. Determination of NFD Entrapment Efficiency Percent (*EE%*)

For determination of NFD entrapped quantity in the nanocomposites, an indirect procedure was utilized as described previously [20]. Particularly, NFD-CTS-PLGA nanocomposites were centrifuged at 4 °C for 2 h at 14,000 rpm (SIGMA 3-30K, Steinheim, Germany). The sedimented nanocomposites were rinsed thrice with distilled water to dismiss the adsorbed drug. Each time, the formed clear supernatant was detached and subjected to filtration (through a 0.45 µm membrane filter) and appropriate dilution before analysis. The quantities of non-entrapped, free NFD were determined in the collected supernatants at 238 nm, utilizing a UV spectrophotometer (Shimadzu UV-1601 PC, Tokyo, Japan). The following equation was employed for computing the *EE%* of NFD (1):

(1) $$EE\%=\frac{total\;drug\;concentration-free\;drug\;concentration\;}{total\;drug\;concentration}\times 100\;$$

#### 2.4.2. Determination of Particle Size and ζ Potential

The mean particle size (z-ave), ζ potential, and polydispersity index (PDI) of NFD-CTS-PLGA nanocomposites were evaluated *via* a Zetasizer Nano ZS dynamic light scattering (DLS) device (Malvern instruments, Malvern, UK). Abrogating the multi-scattering phenomenon was accomplished by diluting the samples with distilled water before the measurement was commenced. To adjust for uncontrolled variability, measurements were run in three replicates and the average values ± SD were recorded. Samples were equilibrated in the measurement chamber for 120 s at a pre-set temperature of 25 ± 2 °C and defined angle to the incident beam of 90°, where three scans were performed on each sample [21].

#### 2.4.3. In Vitro Release Analysis of NFD-CTS-PLGA Nanocomposites

NFD release from the fabricated CTS-PLGA nanocomposites was scrutinized, in triplicate, according to the procedure of membrane diffusion [22] utilizing a Hanson Research, SR 8 Plus model, USP dissolution tester apparatus, type 1 (Chatsworth, CA, USA). Considering the defined *EE%*, nanocomposites dispersions (equivalent to 2 mg NFD) were transferred to 6 cm length, 2.5 cm internal diameter glass cylinders. A dialysis membrane (molecular weight cut off = 12,000 Da), presoaked overnight in the receptor medium, fully enclosed the cylinders on one terminus. The supplied cylinders were secured at the rods of the dissolution apparatus. The release experiments were conducted in 80 mL of phosphate-buffered saline (PBS) pH 7.4, containing 0.1% *w/v* Tween 80 (to fulfill the sink condition) as the release medium [17]. The revolution speed was set to 100 rpm, with the temperature adjusted to 37 ± 0.5 °C over the release study. Definite aliquots (1 mL) were cyclically pulled at programmed time intervals of 0.5, 1, 2, 4, 6, 8, 10, 12, and, finally, at 24 h, where each withdrawal was subrogated with a freshly prepared medium to warrant an unaltered volume. A spectrophotometer fixed at λ_max_ 238 nm was used to determine the accumulative % NFD released, and the average values (±SD) versus time were graphically represented. Analogous, free NFD as a suspension (1 mg/mL, 2 mL in distilled water) was inspected as wellfor in vitro release manner. For analyzing the release kinetics of NFD-CTS-PLGA nanocomposites, the gathered data were fitted into diverse kinetic models and picking of a winning arithmetic model was reliant on the amplitude of the determination coefficients (R^2^).

### 2.5. Optimization of NFD-CTS-PLGA Nanocomposites

Design-Expert^®^ software was employed for election of the optimum nanocomposites formulation through exploiting the desirability function. A formulation with a miniaturized particle size and a maximized *EE%*, as well as *Q*_24 *h*,_ was picked by the optimization process. The target was to identify the formulation composition leading to a desirability value around one. Subsequently, the elected formulation was tailored, appraised, and ultimately compared to the prognosticated responses in an attempt to validate the response surface models.

### 2.6. Morphology of NFD-CTS-PLGA Nanocomposites

The particulate morphology of the optimal nanocomposites formulation was examined utilizing a JEM-1400, Jeol transmission electron microscope (Tokyo, Japan). A drop of the diluted nanocomposites dispersion was settled on a copper grid with a carbon coating. Employing a negative staining procedure, the sample was stained with phosphotungstic acid (2% *w/v*). Thereafter, the samples were explored by the microscope at 80 kV, preceded by drying under ambient temperature [23].

### 2.7. Physical Stability Study of NFD-CTS-PLGA Nanocomposites

The optimum nanocomposites dispersion was predisposed to a stability inspection through storage in a glass vial at 4 °C for three consecutive months. Samples from the optimal nanocomposites formulation were aspirated after fabrication, then after 30, 60 and 90 storage days. The secluded nanocomposites samples were appraised for NFD *EE%*, nanocomposites particle size, as well as ζ potential throughout the storage duration, and analyses were repeated thrice [24].

### 2.8. Aerodynamic Particle Size Characterization

Anderson Cascade Impactor (ACI) (Copley Scientific Ltd., Nottingham, UK), according to the procedure described in appendix 2.9.18 of the European Pharmacopeia and 601 of the United States Pharmacopeia [25,26], was used at room temperature to assess the size distribution of the particle droplets of the emitted drug that would be delivered to rats. The endotracheal tube (ETT) without a cuff, size 5.5 (Zhejiang S. Medical Device Co., Ltd., Zhejiang, China) was inserted into the induction port of the ACI with an airtight seal from one side. The other end of the ETT was connected to the syringe containing the drug. A Brook Crompton vacuum pump (Huddersfield, UK) was employed to provide the vacuum flow through the ACI apparatus (28.3 L/min). An electronic digital flow meter (MKS Instruments, Andover, MA, USA) was used to measure the flow rate. Afterwards, each stage of the ACI was rinsed with a specified volume of acetonitrile, where the amounts deposited on each stage were determined. Utilizing a Copley inhaler testing data analysis software (Copley Scientific, Nottingham, UK), the total dose that would be delivered to the rat (represented as the total mass introduced to the ACI), the amount of the drug that was less than 5 μm aerodynamic diameter reaching the ACI (fine particle dose, FPD), the FPD as a percentage of the total amount reaching the ACI (FPF%), and the mass median aerodynamic diameter (MMAD) of the emitted droplets were determined.

#### Total Emitted Dose

The drug delivery method was assembled in a way that was designed to mimic that of a rat receiving NFD-CTS-PLGA nanocomposites *via* micro-sprayer tubing. Spontaneous breathing was simulated to obtain a flow rate of 28.3 L/min. The breathing simulator setting was chosen to mimic the breathing pattern of healthy rats. The outlet was attached to its standard T-piece and both outlets were connected to the ETT and the ventilation circuit tubing with a tight seal. An electrostatic filter pad, enclosed in a filter holder (Pari GmbH, Starnberg, Germany), was attached next to the breathing machine (inhalation filter). The entire aerosol produced during the inhalation period of a breathing cycle would be entrained on this filter and thus provided a good measure of the total inhaled single dose (the in vitro total emitted dose available for inhalation). A vacuum of 258.3 L/min was drawn through this filter to ensure that it captured the entire dose that was expelled out of the delivery system [27,28]. NFD-CTS-PLGA nanocomposites dispersion (20 mg/mL, 100 µL) was emitted to the ETT and six successive determinations were recorded (*n* = 6). NFD deposited on each filter (left in the ETT and entrained within the tubing) was retrieved by rinsing with acetonitrile. Before rinsing, the drug entrained on the filter was sonicated with acetonitrile. A modified ultra-performance liquid chromatography/tandem mass spectrometry (UPLC/MS/MS) technique was adopted to estimate NFD amounts [29]. The mobile phase, a blend of acetonitrile and 10 mM ammonium acetate (80:20% *v*/*v*), was run at a flow rate of 0.15 mL/min. The column effluent was monitored by employing tandem mass spectrometry in a positive electrospray ionization mode, utilizing 25 eV cone voltage and 15 eV colliding energy. For the quantification process, multiple reaction monitoring (MRM) mode was employed: m/z 347.16 → 315.24 for NFD and m/z 559.34 → 440.33 for atorvastatin (the internal standard, IS), with a dwell time of 0.159 s per transition. The gas temperature was maintained at 400 °C whilst the gas flow rate was 400 L/h. The complete system composed of an Acquity UPLC^TM^ 3100 system (Waters, Milford, MA, USA), a Quattro Premier XE Mass Spectrometer (Waters), Waters Mass Lynx^TM^ software V4.1, and an Acquity UPLC^TM^ Phenomenex Luna^®^ Omega polar C18 column, with a 1.6 µm, 2.1 × 150 mm column (Waters).

### 2.9. Pharmacokinetic Studies

#### 2.9.1. NFD Administration to Rats

A total of 18 male Wistar rats (150–200 g), obtained from the animal facility of the Research Institute of Ophthalmology (Giza, Egypt), were randomly distributed into three groups (*n* = 6 per group). The animals were lodged in polyacrylic cages at a temperature of 22 ± 2 °C and a relative humidity of 55 ± 5% and subjected to a dark/light cycle of 12 h each. Standard diet and tap water was provided to the rats *ad libitum* till the night prior to dosing, where the animals were fasted for 12 h. NFD was administered in a dose of 10 mg/kg [5]. Group 1 received oral NFD suspension (4 mg/mL) in purified water, containing 0.5% *w/v* of sodium carboxymethyl cellulose (Na CMC) through oral gavage, whereas groups 2 and 3 were intratracheally instilled with NFD suspension and the optimized NFD-CTS-PLGA nanocomposites (20 mg/mL), respectively, following anesthesia with ketamine (12.5 mg/kg) and xylazine (1.5 mg/kg) intraperitoneally injected utilizing a Microsprayer^®^ IA-1C system (Penn-Century, Philadelphia, PA) [30]. At scheduled intervals (0.5, 1, 2, 4, 6, 8 and 24 h post-administration), blood samples (1 mL) were withdrawn from the retro-orbital plexus in heparinized tubes to abolish clotting. The plasma was attained by centrifuging the tubes at 3000 rpm for 10 min. The detached plasma was kept at –20 °C till the assay was conducted. The protocol of this study was reviewed and approved by our institutional Animal Ethics Committee of Beni-Suef University which obeys the guidelines of the National Institutes of Health Guide for Care and Use of Laboratory Animals (approval code. REC-A-PhBSU-20013). All procedures for agent administration, blood and tissue collection were in agreement with the Guide for the Care and Use of Laboratory Animals (8th edition) published in 2011 by the United States National Academy of Sciences.

#### 2.9.2. UPLC/MS/MS Operating Conditions

Plasma samples were inspected for NFD, implementing the altered UPLC/MS/MS method described before.

#### 2.9.3. Samples Preparation for Analysis

The frozen plasma samples were kept at ambient temperature for thawing. Plasma samples (100 µL) were blended with 10 µL of IS stock solution (100 µg/mL) then vortex-mixed for 10 s. Two mL ethyl acetate was introduced, and samples were as wellvortexed for 1 min in order to extract the drug. Next, the tubes were centrifuged at 4000 rpm for 10 min. Afterwards, the upper organic phases were conveyed into cleaned glass tubes with a subsequent evaporation till dryness at 30 °C. Dried residues were reconstituted by 300 µL mobile phase and, finally, 10 μL was injected, utilizing the autosampler.

#### 2.9.4. Pharmacokinetic Data Analysis

Estimation of pharmacokinetic parameters from plasma data was executed applying PK Solver software (Excel Add-Ins program) [18,22]. From each rat plasma concentration–time curve, non-compartmental pharmacokinetic modeling was employed for estimation of C_max_ (the maximized NFD concentration, ng/mL), T_max_ (the entailed time for accomplishing C_max_, h), AUC_0–24_, and AUC_0–∞_ (the area under the curve from 0 to 24 and from 0 to infinity respectively, ng h/mL). The trapezoidal rule method was adopted for calculating the areas under the curve. Lastly, F_rel_ (the relative bioavailability, %) for both intratracheal instillations was computed, with respect to oral NFD suspension as a standard.

### 2.10. In Vivo Experimental Study of Bleomycin-Induced Pulmonary Fibrosis in Rats

#### 2.10.1. Animals

Sixty male, Wistar rats weighing 150–200 g were obtained from the animal facility of the Research Institute of Ophthalmology (Giza, Egypt). Two weeks prior to the commencement of the study, the animals were grouped randomly, and lodged in polyacrylic cages. The cages were housed in an air-conditioned, pathogen-controlled room under standard laboratory conditions (temperature 22 ± 2 °C, 12/12 h dark and light cycle and allowed standard forage and tap water *ad libitum*) to permit adaptation to the laboratory environment. Approval of the study was granted by the institutional Animal Ethics Committee of Beni-Suef University (approval code. REC-A-PhBSU-20013). We exerted all efforts to minimize the number of experimented rats and to keep their suffering to a minimum.

#### 2.10.2. Induction of Bleomycin-Induced Pulmonary Fibrosis

Bleomycin was used for the induction of pulmonary fibrosis in all groups, except for the first two groups. Bleomycin sulfate was dissolved in sterilized normal saline (3 U/mL). Briefly, rats were intratracheally instilled with bleomycin sulfate (5 U/kg BW) as previously reported [31], using a Microsprayer^®^ IA-1C system under anesthesia, *via* intraperitoneally-injected ketamine and xylazine.

#### 2.10.3. Experimental Design

The rats were randomly split into six groups (*n* = 10 per group):

Group 1 (control): normal rats were given normal saline *via* intratracheal instillation and the oral route for three weeks.

Group 2 (NFD control): normal rats were orally given NFD (10 mg/kg BW) [5] as a suspension prepared in 0.5% *w/v* Na CMC for three weeks.

Group 3 (bleomycin-induced pulmonary fibrosis): rats were given bleomycin 5 U/kg BW by intratracheal instillation once.

Group 4 (bleomycin + oral NFD): rats were given bleomycin as in group 3 in addition to an oral NFD suspension (10 mg/kg BW) for three weeks.

Group 5 (bleomycin + intratracheal NFD suspension): rats were given bleomycin as in group 3 plus an intratracheal NFD suspension (10 mg/kg BW) for three weeks.

Group 6 (bleomycin + intratracheal NFD-CTS-PLGA nanocomposites): rats were given bleomycin as in group 3 plus intratracheal NFD-CTS-PLGA nanocomposites 10 mg/kg BW for three weeks.

The dose was adjusted every week according to any change in body weight to ensure an accurate dose per kg body weight was given to the rats over the entire period of study for each group.

#### 2.10.4. Lung Sample Collection and Biochemical Assays

At the end of three weeks, the rats were euthanized, and the lungs were removed quickly, cleared from extraneous tissues and blood clots with ice-cold saline. The left lobe of the lung was used for a histopathological examination and a Western blot analysis of TGF-β, GSK-3β, β-catenin, and α-SMA, while the right lobe was frozen at −80 °C for measurements of the hydroxyproline levels, MMP7 activity, and oxidative stress, as well as antioxidant parameters. 

#### 2.10.5. Assessment of Oxidative Stress Markers

##### Estimation of Malondialdehyde (MDA) Level in Lung Tissues

MDA levels were estimated in lung tissues using a colorimetric kit purchased from Biodiagnostic (Giza, Egypt), according to the manufacturer’s instructions.

##### Estimation of Superoxide Dismutase (SOD) Level in Lung Tissues

SOD, an antioxidant enzyme, activities were estimated in lung tissues using a colorimetric kit purchased from Biodiagnostic (Giza, Egypt), according to the manufacturer’s instructions.

#### 2.10.6. Assessment of Fibrotic Markers in Lung Tissues

##### Estimation of Hydroxyproline Levels in Lung Tissues

Hydroxyproline levels were assessed in lung tissues using a rat hydroxyproline ELISA kit (catalogue # E0511Ra), obtained from the Bioassay Technology Laboratory (Shanghai, China), according to the manufacturer’s instructions.

##### Estimation of Matrix Metallopeptidase-7 (MMP-7) Activity in Lung Tissues

MMP-7 activity was assessed in lung tissues, using a rat MMP-7 ELISA kit (catalogue # NBP3-06896), obtained from Novus Biologicals (Centennial, USA), according to the manufacturer’s instructions.

#### 2.10.7. Western Blot Analysis for Detection of TGF-β, GSK-3β, β-Catenin and α-SMA 

In a combination of Tris lysis buffer (400 mM NaCl, 0.5% Triton X-100, 50 mM Tris pH 7.4) and a protease inhibitor cocktail (Biospes, Chongqing, China), rat lung tissues were homogenized for 30 min at 4 °C. Centrifugation (12,000 rpm for 15 min at 4 °C) was employed to remove any residual tissue. To measure the total protein concentration, Bradford method [32] was utilized. Equal quantities of protein (the total protein in each lane was 50 mg) were resolved by 10% SDS-polyacrylamide gel electrophoresis and transferred to a Millipore PVDF membrane (Sigma Aldrich, MO, USA) using semidry transfer methods [33]. The membranes were blocked at room temperature for 1 h with 5% non-fat milk in TBST buffer. Afterwards, the membranes were incubated overnight at 4 °C with primary antibodies. The antibodies used were anti-TGF-β (Elabscience, Wuhan, China; dilution 1:1000), anti-β-catenin (Santa Cruz, Dallas, TX, USA; dilution 1:1000), anti-GSK-3β (Santa Cruz, Dallas, USA; dilution; 1:1000), anti-α-SMA (Elabscience, China; dilution 1:1000), and anti-β-actin antibodies (Santa Cruz, Dallas, TX, USA; dilution 1:5000). The membranes were incubated with alkaline phosphatase-conjugated secondary antibody (Santa Cruz, Dallas, USA, dilution 1:5000) for a 1 h period. A BCIP/NBT substrate detection kit (Genemed Biotechnologies, San Francisco, CA, USA) was employed to visualize bands. As an internal control of protein loading, a Western blot analysis of β-actin was performed. Image J^®^ software (National Institutes of Health, Bethesda, MD, USA) was used to analyze the produced bands in relation to β-actin.

#### 2.10.8. Preparation of Bronchoalveolar Lavage (BAL) 

For collection of BAL, the rat’s trachea was exposed in the neck area and separated from the neighboring tissues. An 18 G needle was inserted into the trachea, and the lung was washed twice using 5 mL sterile saline [34]. The recovered fluid from each rat was used for counting the percentage ratios of lymphocytes, macrophages, and neutrophils to total cell number [35].

#### 2.10.9. Histopathological Examination

The lung tissue samples were fixed in 10% formalin and embedded in paraffin. A histological examination was conducted according to the methods reported in Bancroft and Gamble [36]. The processed samples and the obtained sections were stained with hematoxylin and eosin (H&E), as well as Masson’s trichrome stains. Scoring was performed applying the Ashcroft scale according to Hübner et al. [37].

### 2.11. Statistical Analysis

Results are articulated as the mean ± SD. Statistical significance was determined through a one-way analysis of variance (ANOVA) and then followed by a Tukey’s post-hoc test for multiple comparisons, using SPSS 22 (SPSS, Chicago, IL, USA). The *p* values less than 0.05 were considered statistically significant.

## 3. Results and Discussion

In this study, diverse process variables comprising CTS concentration, PLGA content, surfactant level, organic phase/volume, drug quantity, as well as homogenization rate were investigated employing the emulsion–solvent evaporation method to accomplish optimum fabrication conditions. CTS concentration was maintained at 0.25% *w/v* as an optimal level in all nanocomposites formulations where the particulate size was found to be dramatically enlarged with increment of CTS content beyond this level. PVA was elected as a surfactant, since it represents one of the fittest stabilizers that hampers nanoparticles agglomeration throughout post-preparative steps, as well as reinforces the nanoparticulate yield. Additionally, it is considered as an economical commercialized product with esteemed biocompatibility characteristics [38]. It was attempted at different levels, fluctuating from 0.5 to 1.5% *w/v,* where aggregates were perceived upon absence of stabilizer. Nonetheless, the resultant nanocomposites diameter was reduced, with a marked decrement in *EE%* upon incorporating higher PVA concentrations (˃1.5% *w*/*v*). Moreover, adequate solvent selection for PLGA is an utterly crucial process, since several competing parameters are to be estimated. The solvent/water miscibility can be a determinant factor concerning the nanocomposites shape and drug content [39]. Water miscible solvents, such as acetone and acetonitrile, yielded aggregated composites rather than uniform spheres. On the other hand, water immiscible solvents, such as chloroform and ethyl acetate, implied a diminished solvent diffusion rate in the aqueous medium, signifying a lower *EE%* of the loaded drug. To fulfill all these criteria, a blend of dichloromethane and acetone was utilized for preparing the organic phase. Dichloromethane enabled the assembly of regular spheres through the process of emulsification; meanwhile, it triggered a fast PLGA sedimentation, whereas acetone depicted superiority as a solvent for NFD. Furthermore, the organic phase volume was highly pivotal because it monitored the particulate size, where the increment of organic phase volume was accompanied by a decrease in the particle size (data not shown). In regards to the preliminary trials, the CTS-PLGA nanocomposites were typically tailored with these conditions: 10 mg NFD, 1:4 ratio of organic phase:aqueous phase, and 13,500 rpm homogenization speed for 10 min.

### 3.1. Analysis of the Factorial Design

Statistical designs, such as the full factorial, present a remarkable tool for scrutinizing the integrated effects of causal factors on the nano-cargo features, utilizing the lowest number of experimentations. Contrary to executing sets of independent investigations, such a design permits all the opted preparation variables to be concurrently altered, with a simultaneous assessment of their impacts besides any potential interaction among them on the recorded responses [40]. In the current work, a 3^2^ full factorial experimental design, as implemented in the Design-Expert^®^ software, was employed and statistically evaluated. Each variable levels were established, based on the outcomes of the preliminary studies, in addition to the feasibility of fabricating NFD-CTS-PLGA nanocomposites through these values. The polynomial quadratic model was designated as the bestfit model regarding the three measured responses as compiled in Table 3. Adequate precision appraised the signal-to-noise ratio, for emphasizing the model aptitude to navigate the design space [41]. A ratio higher than four is eligible, which was remarked in all observed responses, Table 3. Additionally, the predicted R^2^ was estimated to measure the model goodness to envisage a dependent response value. The values of the predicted and adjusted R^2^ are favored to be in a close harmony (within ∼ 0.2) to each other [42]. In the present study, it is noteworthy that the values of the predicted R^2^ were in line with those of the adjusted R^2^ in the three responses.

#### 3.1.1. Effect of Formulations Variables on *EE%*

The capability of the tailored CTS-PLGA nanoplatform to encapsulate appreciable quantities of NFD is substantial for its potential exploitation as a pulmonary targeting paradigm. The *EE%* values of the assembled NFD-CTS-PLGA nanocomposites oscillated from 53.62 ± 2.54 to 77.18 ± 2.75% at various levels of the two explored causal factors, as profiled in Table 2. This relatively high *EE%* for the copolymeric nanocomposites could be ascribed to the hydrophobic trait of NFD that had minute affinity for the aqueous medium (∼10 µg/mL in water at 37 °C) [43] and hence, resorted to immigrate to the organic compartment. Also, such plausible *EE%* values might be accredited to a possible electrostatic interaction between the cationic CTS polymer and the weakly acidic drug, NFD (pKa = 3.93) [43]. ANOVA testing for the detected *EE%* data disclosed best fitting of the data through the quadratic model that was based on its highest R^2^ value. The model F-value was assigned to be 279.49 and it was statistically significant (*p* ˂ 0.0001). Consequently, the *EE%* values of NFD-CTS-PLGA nanocomposites were significantly influenced by both causal factors (*p* ˂ 0.0001). Equation (2) is the regression equation allying the impact of PLGA (*X*_1_) and PVA (*X*_2_) concentrations on the *EE%* (*Y*_1_), as a function of the coded values.

(2) $$EE\%=+65.91+7.29X_{1}-2.30X_{2}+0.11X_{1}X_{2}-4.96X_{1}^{2}+0.09X_{2}^{2}$$

**Figure 1:**
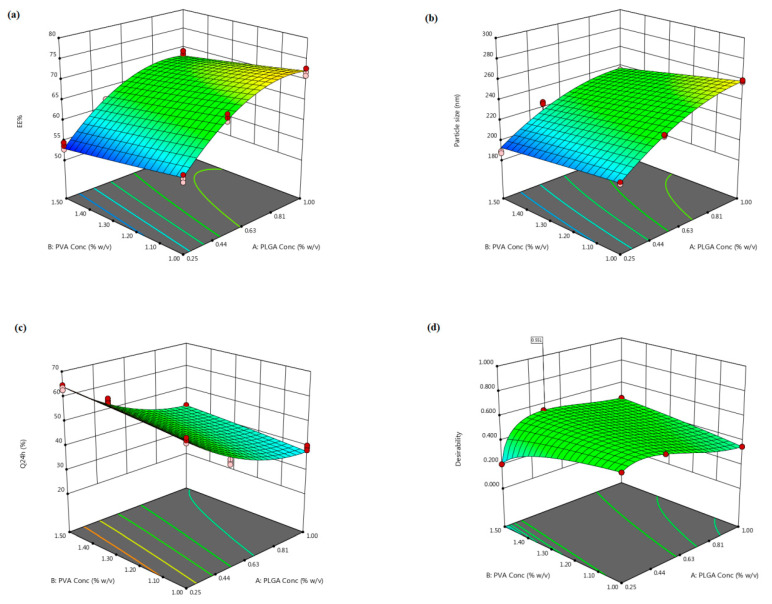
The three-dimensional plot depicting the response surface to the impact of PLGA (*X*_1_) and PVA (*X*_2_) concentrations on (**a**) NFD *EE%* (*Y*_1_), (**b**) nanocomposites particle size (*Y*_2_), (**c**) *Q*_24 *h*_ (*Y_3_*) as well as (**d**) desirability of the tailored nanocomposites.

According to the regression coefficients, PLGA concentration (*X*_1_) exerted an obvious significant synergistic influence on the *EE%* values, whereas PVA concentration (*X*_2_) evinced a significant antagonistic impact, *p* ˂ 0.0001. The effect of PLGA (*X*_1_) and PVA (*X*_2_) concentrations on NFD *EE%* within the nanocomposites is displayed as 3D response surface plot in Figure 1a. The positive impact of PLGA concentration (*X*_1_) on the *EE%* values might be succumbed to its direct influence on viscosity, where raised PLGA levels could confer a viscid diffusional barrier that curtailed NFD permeation into the external aqueous medium and, hence, strikingly higher *EE%* values were elicited [44]. Also, the greater the viscosity, at elevated PLGA concentrations, the lesser the net shear stress, which could generate larger particulate sizes. Larger-sized nanocomposites would afford adequate space for NFD molecules to be ensnared [45]. Jose et al. [46] claimed analogous outcomes upon assessing the characteristics of carboplatin-laden Tween 80-caoted PLGA nanoparticles.

Contrarily, raising PVA concentration (*X*_2_) was escorted with a simultaneous decrement in the *EE%* values of NFD, Figure 1a. This significant decline could be accredited to the miniaturized interfacial tension that promoted rapid partitioning of NFD molecules from the organic phase, with solubilization as micelles into the aqueous medium during the emulsification process at higher PVA concentrations [47]. Additionally, the reduction in particle size, triggered by amplified PVA levels, could arouse a lower entrapped NFD. This observation is in harmony with those accomplished by earlier studies [46,48].

#### 3.1.2. Effect of Formulation Variables on Particle Size

Particle size is a fundamental parameter since it could directly influence the drug release, physical stability, biodistribution, as well as cellular uptake. Nanosized particulate range possesses a prosperous accessibility within the body, as it is freely conveyed through the systemic circulation to various bodily organs [49]. As summarized in Table 2 and illustrated in Figure S1, (Supplemental File), the average particle size of the fabricated NFD-CTS-PLGA nanocomposites spanned from 188.20 ± 10.34 to 280.41 ± 11.63 nm. PDI was recorded for appraising the homogeneity of the nanoparticulate dispersions. A monodispersed nanopopulation is denoted with a zero value, whilst a value of one implies immensely polydispersed particles [24]. The PDI of the assembled nanoformulations varied between 0.235 and 0.473, specifying a restricted size distribution with notable homogeneity, Table 2. Quadratic model divulged the highest significant mean squared value above the residual error (*p* ˂ 0.0001), with an insignificant lack of fit (*p* = 0.4960) and, thus, it was applied for particle size analysis. Equation (3) demonstrates the quantitative influence of the two independent variables on the particle size of NFD-CTS-PLGA nanocomposites in coded values.

(3) $$Particle\;size=+232.26+24.24X_{1}-8.68X_{2}-1.94X_{1}X_{2}-10.96X_{1}^{2}-0.25X_{2}^{2}\;\;$$

The inspected causal factors disclosed a significant outcome on the mean particle size among the different nanocomposites (*p* < 0.0001). As regards to PLGA concentration (*X*_1_), the ANOVA results manifested an unfavorable synergistic impact on the compiled particle size (*p* < 0.0001). As has already been outlined, the snowballed organic phase viscosity, presented *via* upgrading PLGA levels, could handicap fast dispersibility of PLGA solution through the aqueous medium, with attenuation of the overall shearing forces, therefore, promoting assembly of larger-sized droplets that constituted bigger nanocomposites following the organic solvents evaporation [45]. Another hypothetical elucidation could be admitted to the notion that, with rising PLGA concentration, PVA was possibly disabled to entirely enclose the droplets’ surface, with subsequent coalescence of droplets during the organic solvents removal, provoking aggregated nanocomposites and, hence, a large particulate size was conceivable [46]. Moreover, higher PLGA levels might induce polymer–polymer interactions, thereby extra polymeric domains could persist connected during the diffusion of the solvent into the aqueous medium [50]. Emami et al. [51] reported that the higher the PLGA content, the higher the particle size would be, thus implying compatible results.

With respect to PVA concentration (*X*_2_), it had a favorable negative impact on the average particulate size of the nanocomposites (*p* < 0.0001). This finding might be ascribed to the aptitude of more PVA to be aligned at the aqueous/organic interface, with an infallible diminution of the interfacial tension, leading to a remarkable increment in the shearing power at a fixed energy density throughout the emulsification process, which could fortify the assemblage of smaller emulsified droplets [52]. This result is in line with those presented by previous arts [14,46,48,52]. On the other hand, the aforementioned finding was contradictory with other reports [53,54] that narrated enlarged particle sizes linked with greater PVA levels, which were surrendered to PVA gelatinization due to robust hydrogen bonding through hydroxyl moieties among intra/inter PVA molecules, provoking an increased particle size coupled with decreased *EE%*. However, this discrepancy could be argued in terms of the dissimilarity in PVA levels employed in these studies.

#### 3.1.3. Effect of Formulation Variables on *Q*_24 *h*_

**Figure 2:**
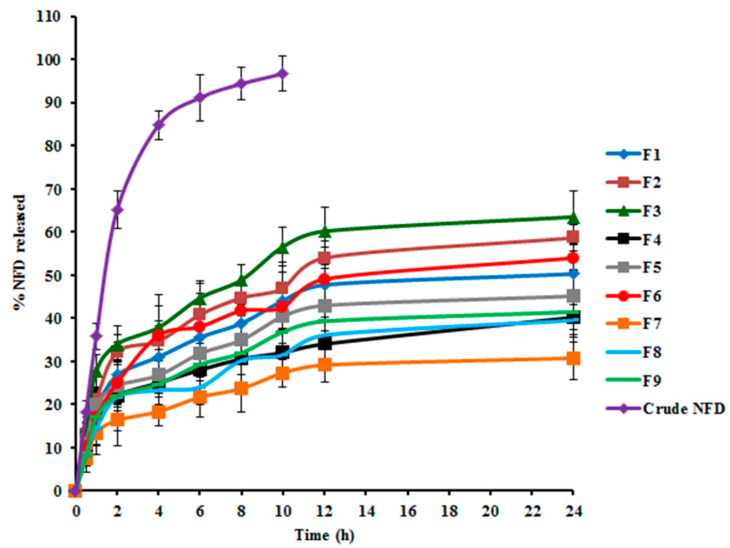
NFD in vitro release profiles from NFD suspension and various NFD-CTS-PLGA nanocomposites formulations.

There are diverse factors influencing the hydrophilic traits and degradation rates of a polymeric delivery system, which consequently inaugurate the drug release from the tailored nano-cargo, such as molecular weight and structure of the enrolled polymers, array and size of the nanoparadigm, physical characteristics of the delivery matrix, loaded drug concentration, and the release medium pH [55]. The release profiles of NFD from the assembled CTS-PLGA nanodispersions, as well as its aqueous suspension in PBS pH 7.4, are graphically represented in Figure 2. Within 10 h, 96.67 ± 4.02% NFD release ensued from plain drug suspension, whereas the *Q*_24 *h*_ from CTS-PLGA nanocomposites fluctuated from 30.50 ± 2.37 to 63.65 ± 2.72%, as revealed in Table 2, enlightening a sustained release behavior from the fabricated nano-cargo. Such slower NFD release might be explicated in the light of the relatively high molecular weight of the employed PLGA copolymer (50:50, molecular weight 85,000 Da) [56]. Actually, there is a direct correlation between the polymer molecular weight and its glass transition temperature (Tg), wherein an increment in molecular weight typically raises the Tg, which predetermines a rubbery or glassy feature of the matrix beyond or beneath it, respectively. Concerning high molecular weight PLGA nanocomposites, a slow water hydration, owed to higher polymer lipophilicity, could evoke shifting of the Tg to a higher temperature as a result of annulling the plasticizing impact of water, which rendered the copolymeric chains less mobile and hence less vulnerable to degradation [57]. Additionally, the degradation pattern of a polymeric system is not only reliant on the polymeric molecular weight but also on the lipophilic/hydrophilic ratio of the copolymer. The higher the polymeric hydrophilicity, the faster the degradation would be. The polymeric hydrophilicity is dependent on the ratio of amorphous to crystalline domains, which is related to the copolymeric structure. PLGA copolymers made from poly-D, L-lactic acid and poly glycolic acid, utilized in this study, are amorphous in nature, whereas those composed of L-poly lactic acid and poly glycolic acid possess the crystalline trait. Yet, lactic acid is less hydrophilic than glycolic acid, which donates the lactide-enriched PLGA copolymers relative hydrophobicity that, in turn, hinders the biodegradation process [55,56]. Moreover, considering the CTS matrix that tagged the nano-cargo, an additional barrier for drug diffusion could be signified with consequent impedance of NFD release [22]. Notably, NFD release profiles from various CTS-PLGA nanocomposites were apparently biphasic processes, with an early burst (first 2 h) supervened by a prolonged release. The burst–release might be allied with the surface-entangled drug molecules that were instantaneously desorbed upon contacting the release medium. Another plausible mechanism could be assigned to the recorded small particulate size, which might create a shorter diffusional path for the matrix-trapped NFD, causing an initial rapid release [58]. Thereafter, the diffusion of the entrapped NFD across the polymeric matrix during the second phase is evidenced from the retarded release rate [59]. Such observations are endorsed with earlier literature reports declaring biphasic release pattern of polymeric nano-cargos [60,61]. The recommended quadratic model turned out to be statistically significant on residual analysis, as well as on ANOVA (adequacy/precision ratio was 47.13), pointing out an adequate signal. Equation (4) interrelates the *Q*_24 *h*_ to both causal factors in coded values.

(4) $$Q_{24\;h}=+47.03-10.57X_{1}+2.56X_{2}-0.31X_{1}X_{2}+4.92X_{1}^{2}-0.27X_{2}^{2}\;\;\;\;\;\;$$

The investigated terms were associated with a significant impact on the *Q*_24 *h*_ parameter, *p* < 0.0001. As demonstrated in Figure 1c, PLGA concentration (*X*_1_) exerted a significant negative influence on the average *Q*_24 *h*_, *p* ˂ 0.0001. Such antagonistic effect could be imputed to the enlarged particle size prevalent at higher PLGA levels, as previously indicated, which presented a smaller surface area/volume ratio disclosable to the release medium and, thus, encumbered NFD release. Additionally, increasing PLGA concentration could hamper NFD release *via* diffusion [46]. Moreover, the drug release rate from biodegradable systems is reliant on the drug affinity to polymeric matrices. Expectedly, hydrophobic drugs, as NFD, could have a greater affinity for higher molecular weight PLGA copolymers and thus, NFD was slowly partitioning out of the matrix, manifesting a considerable sluggish release at higher PLGA levels [56]. This finding coincides with that of Sharma et al. [62] who narrated lessened drug release rates with raising PLGA concentration upon fabrication of such biodegradable nano-cargo for parenteral paclitaxel delivery.

It is worth mentioning that PVA concentration (*X*_2_) divulged a significant synergistic action on the NFD *Q*_24 *h*_ (*p* ˂ 0.0001). This positive interrelation between PVA concentrations and the *Q*_24 *h*_ could be accounted for by the particulate size, since the drug release percentage, throughout the experimentation period, is inversely proportional to the nanocomposites’ diameter. Hence, smaller composites procured at escalated PVA concentrations could diminish the diffusional interspace for the drug, with consequent snowballing of NFD release as a function of time [52]. As noted before, this phenomenon could be claimed to augmented NFD partitioning between the lipid matrix and the aqueous medium, pursuant to ameliorated drug solubility at higher surfactant levels. It is noteworthy that our data are parallel to that outlined by other studies [18,47,51].

Arithmetical analysis of NFD release data elucidated congruity of the drug release from almostassembled nanodispersions with Higuchi release kinetics, implying a diffusion-controlled pattern. Numerous trails reported that drug-laden polymeric nano-cargo manifested a sustained release manner conformed to Higuchi’s square root equation [60,62]. Additionally, NFD release behavior from CTS-PLGA nanocomposites was further scrutinized by applying the Korsmeyer–Peppas model. The computed *n* values among various formulations vacillated from 0.57 to 0.68, demonstrating the non-Fickian diffusion i.e., anomalous release fashion (0.5 < *n* < 1), wherein drug diffusion might be concomitant with the polymeric membrane erosion [49]. Generally, PLGA copolymers are susceptible to bulk degradative processes through hydrolytic scission of the matrix ester linkage. The cleaved acidic monomers (glycolic and lactic acids), as well as oligomers, spur the parent polymer degradation, which is recognized as autocatalysis. Thus, the release of the loaded drug from PLGA matrices could occur *via* a diffusion-cum-degradation primed mechanism [63].

#### 3.1.4. Optimal Formulation Identification

The optimization of a tailored paradigm is commonly designated for demarcating the optimal variables levels, wherein a robustly qualified pharmaceutical product could be fulfilled [21]. Thus, the desirability approach was pursued, applying Design-Expert^®^ software for the election of the optimum formulation among the nine elaborated dispersions, based on the 3^2^ full factorial design. The desirability function constitutes the modeling of various physicochemical characteristics, as a geometrical estimate, in order to confer a model having maximal value, which can be existed into the design space for procuring the eligible formulation [18]. The desirability constraints for the optimized formulation (maximum *EE%* and *Q*_24 *h*,_ with minimum particle size) were estimated with net desirability index of 0.551, Figure 1d. The optimal formulation was assembled utilizing 0.52% *w/v* PLGA and 1.5% *w/v* PVA, besides 0.25% *w/v* CTS; this formulation disclosed *EE%* of 61.81 ± 3.41%, particle size of 226.46 ± 7.21 nm, and *Q*_24 *h*_ of 50.40 ± 3.83%. For assessing the optimization process validity, a comparison between the observed and prognosticated responses was carried out, and the results are indicated in Table 4. The great congruity between the prognosticated and observed values manifested as a small % prediction error that equivocated between 1.71 and 3.27% for the different responses, suggesting the adequacy as well as validity of the proposed mathematical models for scrutinizing the explored responses. Therefore, this formulation was chosen for further inspections. 

### 3.2. Transmission Electron Microscopy

**Figure 3:**
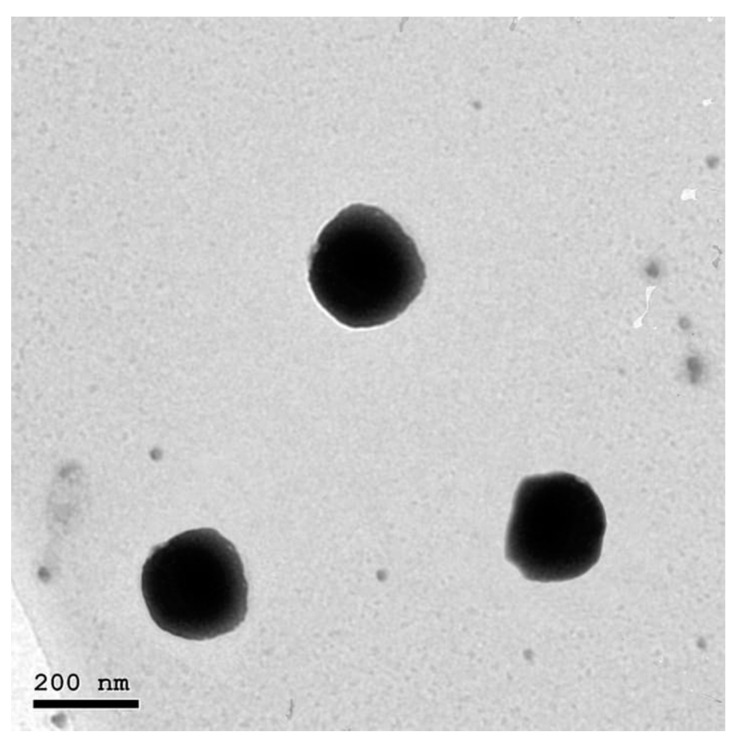
Transmission electron photomicrograph of the optimum NFD-CTS-PLGA nanocomposites.

TEM imaging was employed for the visualization of the morphological aspects of the optimized NFD-CTS-PLGA nanocomposites, as elucidated in Figure 3. The inspected composites were nanostructured, non-aggregating, and nearly spherical, possessing a unimodal size distribution without any NFD crystals noted. Morphological speculation was well concurred with the recorded particle size values that were measured *via* DLS. Additionally, monodispersed nanocomposites could demonstrate the presence of adequate surface charges that provoked well segregation of the fabricated nano-cargo which was further emphasized by the determined positive ζ potential value.

### 3.3. Stability Study of NFD-CTS-PLGA Nanocomposites

**Figure 4:**
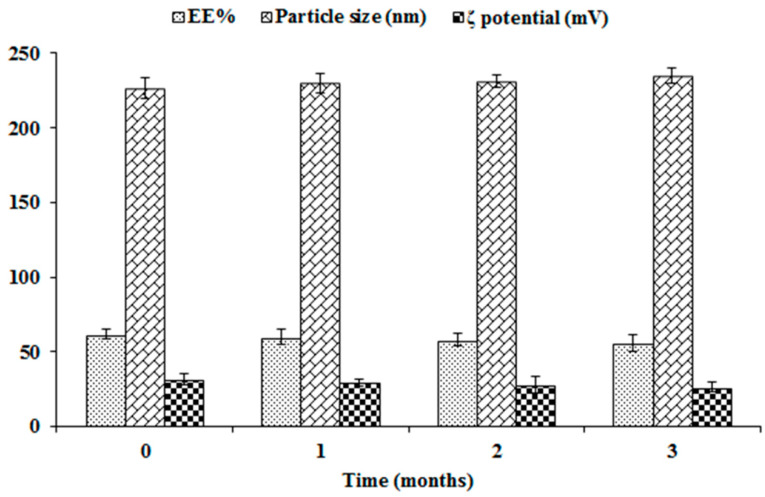
Influence of storage on *EE%*, particle size, and ζ potential of the optimal NFD-CTS-PLGA nanocomposites.

The EE%, particle size and ζ potential were measured over three storage months at 4 °C for appraisal of the physical stability of the optimal formulation. As clarified in Figure 4, the optimized formulation evinced insignificant changes in *EE%*, particle size, as well as ζ potential throughout the study period (*p* > 0.05). A plausible explanation for such high stability could be correlated to presence of competent levels of PLGA, CTS, as well as PVA as a stabilizer. Indeed, the ζ potential demonstrates a good index for nanoparticulate stability. Since it represents an estimate of the overall particles charge, as such, the greater the absolute ζ potential values, the higher the quantity of surface charges. Virtually, a minimal ζ potential of ± 30 mV could trigger a physically stabilized nanodispersion solely *via* electrostatic repulsion [21,47]. In the current work, the average ζ potential of NFD-CTS-PLGA nanocomposites was +31.63 ± 3.72 mV, due to surface functionalization with positively charged CTS, proposing localization of this polysaccharide amino groups onto the surface of the assembled nanocomposites. In addition to the greater imparted stability by such elevated modulus value, the nanocomposites’ cationic corona could promote retention and adhesion at the mucosal surfaces, besides ameliorated absorption of the nano-cargo [22]. 

### 3.4. Aerodynamic Particle Size Characterization

Particulate size is deemed as a substantial parameter in the aerosolization process, since the minimal the particulate size the higher nanoparticles number would accommodate within the micron-sized aerosol droplets, thus, ameliorated deeper drug delivery through the lung could be accomplished due to raised diffusional mobility. An additional merit of the smaller-sized particles is that enhanced drug absorption rate might be occurred *via* provoking even drug distribution [64]. ACI represents the most commonly utilized equipment for measuring the particulate size, based on aerodynamic diameter, as well as the deposition behavior of the conveyed nano-cargo through pulmonary route [65]. Actually, it comprises eight stages, which are oriented in such manner that enlarged particles with adequate inertia could impact on a certain collection plate stage upon passing of the aerosol stream, whilst smaller particulates, having insufficient inertia, would be lifted *via* the air stream, with passage into the subsequent impacting stage [66]. According to our results, the optimized NFD-CTS-PLGA nanocomposites were mostly accumulated and deposited between stages 5–7, and these stages represent the terminal bronchiolar and alveolar regions [67]. The aerodynamic characteristics of the nanocomposites are denoted in Table 5. The MMAD and FPF of the nanocomposites were 1.12 ± 0.28 μm and 80.48 ± 8.46%, respectively. From the data, it appeared that the tailored nanocomposites were able to efficiently deposit to the lungs. 

### 3.5. Pharmacokinetic Studies

**Figure 5:**
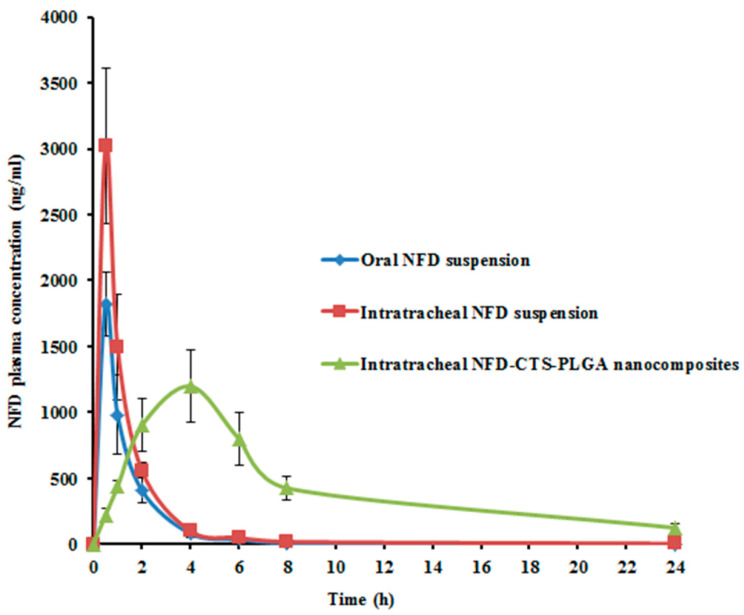
Mean NFD plasma concentrations in rats following administrations of oral and intratracheal suspensions as well as intratracheal optimized nanocomposites. Each value denotes the mean ± SD (*n* = 6).

In vivo pharmacokinetic investigations were conducted for NFD quantification, following the intratracheal instillation of the optimized NFD-CTS-PLGA nanocomposites, compared to either the oral or intratracheal NFD suspensions. The average plasma NFD concentration–time profiles following administrations of the various formulations, are designated in Figure 5, and the analogous pharmacokinetic parameters are recorded in Table 6. NFD dose for the triple treatments was allotted to 10 mg/kg of the animals’ body weight. Both oral as well as pulmonary administrations were of adequate tolerability to the rats. The AUC_0–∞_ and C_max_ of NFD in plasma were 2589.53 ± 178.83 ng h/mL and 1820.76 ± 240.52 ng/mL, 4035.03 ± 630.82 ng h/mL and 3020.23 ± 590.47 ng/mL, as well as 9535.33 ± 940.32 ng h/mL and 1198.22 ± 270.13 ng/mL after administrations of oral and intratracheal suspensions, as well as intratracheal nanocomposites, respectively. The T_max_ of the oral and intratracheal suspensions, as well as the intratracheal nanocomposites administrations, were 0.5, 0.5 and 4 h, respectively. The T_1/2_ was 2.03 ± 0.31 h on peroral administration whereas it was 2.42 ± 0.14 h and 6.66 ± 0.98 h for intratracheal suspension and intratracheal nanocomposites, respectively. The mean residual time (MRT) for the intratracheal nanocomposites was 3.96- and 3.49-fold greater than the correspondent mean values for the oral and intratracheal suspensions, respectively (*p* < 0.05), as listed in Table 6. A slow and prolonged NFD absorption from the intratracheal nanocomposites *via* the lung mucosa was clarified by the dawdled T_max_ alongside extended T_1/2_ and MRT, which were consistent with the outcomes of the in vitro release experimentation earlier. As compared to the oral suspension, the F_rel_ of NFD from the intratracheal suspension was circa 155.82%, and it was 368.23% for the intratracheal nanocomposites. The aforementioned findings revealed superior NFD absorption following pulmonary administration of CTS-PLGA nanocomposites, which might be attributed to the ensuing mechanisms: (i) eschewing the hepatic first-pass metabolism attendant with peroral NFD administration; (ii) smaller particulate size of the assembled nano-cargo, where it was informed that particle diameter reduction beneath 500 nm provoked enhanced drug deposition within all lung regions, mostly due to the ameliorated diffusion mobility [64]; (iii) extended circulatory time and elimination half-life traits, owed to NFD embedding into CTS-PLGA nanocomposites that might protect the drug from enzymatic degradation; (iv) snowballed penetration of the nanocomposites formulation that represents a robust permeation enhancer as a result of the dual impact of hydrophobic and hydrophilic domains, besides the penetration-enhancing aptitude of CTS [22]. CTS displays its permeability-enhancing potential through opening the tight junctions among epithelial cells, in addition to consolidating the paracellular transporting mechanisms and (v) the intense CTS bioadhesive properties, which could lengthen the residence time of the nanocomposites on the pulmonary mucosal surfaces and, hence, lower their exhalation. CTS elicits its mucoadhesive features *via* interaction among its positively-charged amino groups with negatively-charged sialic acid moieties of the mucosal epithelial cells’ glycoproteins [68]. These results are in close agreement with those introduced by Ahmed et al. [69], who developed catechin hydrate-laden CTS-PLGA nano-cargo for lung cancer treatment, with augmented pharmacokinetics and antitumor characteristics. The obtained results implied that NFD-CTS-PLGA nanocomposites could be deemed as a felicitous pulmonary delivery system.

### 3.6. In Vivo Experimental Study of Bleomycin-Induced Pulmonary Fibrosis in Rats

Idiopathic pulmonary fibrosis (IPF) is a serious disease resulting in a high incidence of mortality, with an average survival time following diagnosis of about 2–5 years. As the pathogenesis of IPF is not fully recognized, effective therapeutic options are limited. Although the emergence of two novel drugs, pirfenidone and nintedanib, may help to tackle IPF, unfortunately they are unable to entirely cease it [70]. This demonstrates that we still have a challenge in the complete recovery of IPF, with the need for the detection of more competent therapies.

Studies have been done to improve the pharmacological tools used to modify the activity of T- and L-type calcium channels, due to their role in various cellular and organ functions, and many of these techniques are now crucial in clinical practice. It was demonstrated that calcium oscillations are vital to the growth factor-stimulated synthetic function of pulmonary fibroblasts [71]. Furthermore, Tanaka et al. [34] anticipated that repeated inhalation of felodipine, or other dihydropyridine calcium channel blockers, could be promising candidates for reducing the serious bleomycin side effects in cancer patients. Even if felodipine or other calcium channel blockers were supplied intratracheally in bleomycin-induced pulmonary fibrosis, they improved the lower portions of the lung.

NFD is identified as a highly selective L-type calcium channel blocker and is recognized to have numerous promising pleiotropic effects in several animal models, such as atherosclerosis, aortic aneurysmal formation, and diabetic nephropathy models [72]. Also, Mukherjee et al. [5] mentioned that NFD seems to be the final participant in the series of ionic conductance alterations which cause calcium oscillations. Additionally, this L-type blocker is well tolerated, well defined, quite economical, and has already acquired FDA approval for use in clinical practice for the treatment of a wide range of cardiovascular disorders. Moreover, Vriens et al. [73] reported that the beneficial effects of L-type calcium channel blockers may arise by other low-affinity molecular targets; for example, micromolar concentrations of calcium channels blockers have resulted in the stimulation of the transient receptor potential melastatin-3 (TRPM3) channel. Furthermore, it was observed that NFD dampened the fibrotic changes and the decline in lung function in the pulmonary fibrosis model that was induced by bleomycin [5].

Indeed, bleomycin has been the most commonly applied model of lung fibrosis in experimental animal models. There are several routes of its delivery, such as intratracheal, intraperitoneal, subcutaneous, as well as intravenous, however, intratracheal administration of bleomycin is the most frequent route. Bleomycin provokes direct cell injury *via* the induction of DNA strand breaks, free radicals generation, and oxidative stress stimulation [74]. Accordingly, the present study was steered to evaluate a new pharmaceutical preparation of NFD in the CTS-PLGA nanocomposites form, in comparison with the conventional NFD, using a bleomycin-induced lung fibrosis animal model which aimed to improve its pharmacokinetics and pharmacodynamics that may result in appreciated effects for tackling IPF.

The development and severity of IPF are influenced by oxidative stress and an oxidation/antioxidation imbalance [75]. Reactive oxygen species (ROS) are known to cause an augmentation in lipid peroxidation. MDA is a reactive carbon molecule that is applied as a lipid peroxidation biomarker. On the other hand, SOD and GPx are endogenous enzymes that play a crucial part in the cellular defense mechanisms against oxidative damage. Endogenous antioxidant enzymes abolish ROS such as hydrogen peroxide and inhibit the production of hydroxyl radicals [76]. In the current study, bleomycin resulted in oxidative and anti-oxidant disturbances, evidenced by the significant increase of MDA levels and significant decline of SOD activities. This is in accordance with Shariati et al. [77] who declared that bleomycin is known to cause oxidative damage in the lungs. Hence, scavenging of free radicals appears to be decisive for disease management. It is interesting in the present work to find intratracheal NFD nanocomposites having a pronounced, significant effect on the repression of oxidative stress and enhancing the anti-oxidant activity to a greater extent than the oral and intratracheal NFD suspensions, evidenced by the more declined MDA level and enhanced SOD activity, Table 7.

Collagen and other extracellular animal proteins contain L-hydroxyproline (L-Hyp), a non-proteinogenic and non-essential amino acid. L-Hyp is one of 18 types of amino acids that are found in mammalian collagens, and represents a key component of collagen (elastin contains approximately 1%). L-Hyp is also imperative for collagen synthesis and stability. As a result, L-Hyp is a distinctive amino acid and a beneficial marker for determining the collagen content in IPF tissues [78]. Therefore, L-Hyp has enticed growing attention as a significant biomarker of IPF.

Some matrix metalloproteinases (MMPs) such as MMP-1, MMP-2, MMP-3, MMP-7, MMP-9, and MMP-13 are vastly expressed in IPF, where they play different roles in the outcome of fibrosis; however, the precise mechanisms are not well registered. MMP-7 has already been proposed to exert an essential role in experimental and human lung fibrosis, thus underscoring the dynamic control of the extracellular matrix (ECM) and its restoration processes in the lung [79]. In the present experiment, the intratracheal NFD nanocomposites succeeded in dampening the increase in L-Hyp and MMP-7 levels caused by bleomycin, which was superior to the effect manifested by both the oral and inhaled NFD suspensions, Table 7. 

Fibroblasts are essential mediators of the lung structural changes that arise in IPF. The principal function of fibroblasts is the secretion and storing of cytokines and connective tissue proteins. They respond to numerous growth factors and cytokines, including TGF-β. The classic pathological fibroblast phenotype is the myofibroblast, which has been defined in IPF. Myofibroblasts are recognized by the de novo production of alpha smooth muscle actin (α-SMA), and are avidly synthetic for collagen and other ECM components. Myofibroblasts’ presence in fibrotic lesions corresponds with the progress of active fibrosis in experimental animal models of fibrosis, and their existence, as well as location, to fibrotic foci in human disease is linked to disease progression [80].

Mukherjee et al. [71] verified that TGF-β1-mediated changes of calcium are attributed to both the influx of external calcium, as well as the release of internally sequestered calcium. Furthermore, TGF-β1 is thought to have a pivotal role in the pathogenesis of IPF, specifically in relation to aberrant fibrosis. TGF-β1 seems to enhance collagen synthesis, as well as promote the transition of fibroblasts into myofibroblasts. It was also stated that TGF-β promoted the expression of the mesenchymal marker α-SMA, with a parallel upregulation of transcriptionally active β-catenin in the epithelial cells of the lung. Inhibition of β-catenin reduced the TGF-β-induced α-SMA expression in these cells [4]. Mukherjee et al. [5] revealed that NFD abrogated the bleomycin-induced increase in α- SMA and collagen fiber deposition, as well as the content of soluble and insoluble collagen. 

**Figure 6:**
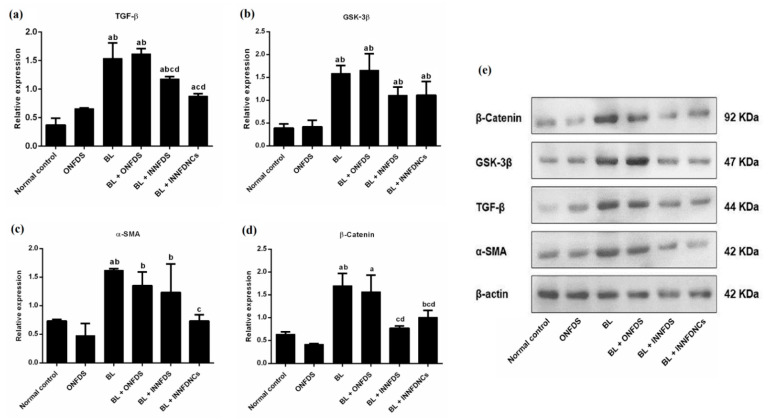
Lung protein expression of TGF-β, GSK-3β, α-SMA, and β-catenin. (**a**) TGF-β, (**b**) GSK-3β, (**c**) α-SMA, (**d**) β-catenin and (**e**) Western blot bands of studied proteins versus β-actin. Values are means ± SD, *n* = 10 for each group. ONFDS: oral nifedipine suspension; BL: bleomycin; INNFDS: inhaled nifedipine suspension; INNFDNCs: inhaled nifedipine nanocomposites; TGF-β: transforming growth factor beta; GSK-3β: glycogen synthase kinase-3 beta; α-SMA: alpha smooth muscle actin (^a^ *p* < 0.05 versus normal control, ^b^ *p* < 0.05 versus ONFDS, ^c^ *p* < 0.05 versus BL, ^d^ *p* < 0.05 versus BL + ONFDS.

Moreover, it was first established that IPF lungs showed nuclear accumulation in alveolar epithelial type 2 cells (AEC2) of β-catenin. Then, several Wnt ligands such as glycogen synthase kinase-3β (GSK-3β) and β-catenin were detected to be greatly expressed in hyperplastic AEC2 and bronchiolar epithelial cells, demonstrating that the Wnt/β-catenin signaling pathway is generated in the epithelium during IPF progression. At the early stages of injury in alveolar epithelial cells, the activation of β-catenin may occur as a challenge for repair and regeneration; however, sustained activation may motivate inflammation and fibrotic alterations in the lung [81]. In harmony with the aforementioned, the NFD nanocomposites in the current experiment surprisingly resulted in a significant downregulation of TGF-β, α-SMA, and β-catenin with eminent impact when compared to the other treatments, Figure 6.

GSK-3β regulates numerous crucial signaling pathways throughout the fibrotic process, which has been demonstrated to play an imperative role in the protection of initial inflammatory injury, the stimulation of related effector cells, epithelial-to-mesenchymal transition (EMT), and ECM accumulation. GSK-3β has also attracted considerable attention as a key regulator of aging and lifespan. As a result, a deep understanding of GSK-3β’s activities in the fibrotic response may aid in the discovery of appropriate targets for anti-fibrotic clinical therapies. Also, GSK-3β is tangled in TGF-β1–dependent differentiation to myofibroblasts, as well as in EMT. Additionally, GSK-3β inhibition has resulted in suppressing α-SMA protein levels in primary human lung fibroblasts [82].

**Figure 7:**
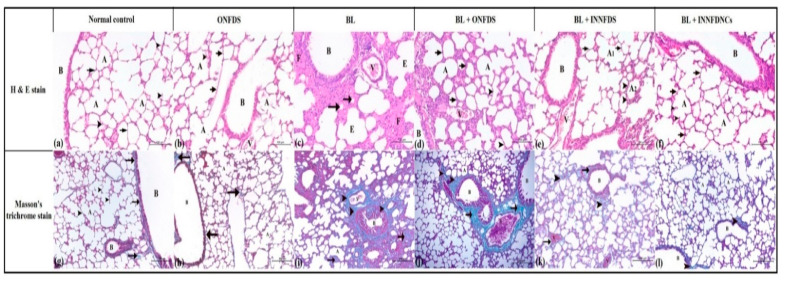
Histopathological features of lung tissues from all groups after three weeks of treatment. (**a**–**f**): lung tissues stained with hematoxylin and eosin (H & E). Magnification ×100. (**a**)—normal control group showed normal pulmonary tissues with normal bronchus and bronchiole (B). The lung alveoli (A) appeared normal with normal and thin alveolar epithelium and inter-alveolar septum (arrow). The alveoli, containing alveolar macrophages (arrow heads). (**b**)—oral NFD suspension-treated group showed normal pulmonary tissues with normal bronchus and bronchiole (B). The lung alveoli (A) appeared normal with normal and thin alveolar epithelium and inter-alveolar septum (arrow). The alveoli, containing alveolar macrophages (arrow-heads). (**c**)—bleomycin group showed degenerative changes in the epithelial lining of bronchioles (B) and alveoli (A). The alveoli in the lung showed atelectatic (atrophied) alveoli (arrow) and the others suffered from emphysematous changes (E). Note, thickening of the inter-alveolar septum (F) and congestion of the pulmonary blood vessels (V). (**d**)—oral NFD suspension + bleomycin group showed few degenerative changes in the epithelial lining of bronchioles (B) and alveoli (A). The majority of the inter-alveolar septum suffered from thickening (arrow), while few septum appeared normal (arrow-heads). The pulmonary blood vessels showed congestion (V). (**e**)—inhaled NFD suspension + bleomycin group showed few degenerative changes in the lining of the epithelium of bronchioles (B). The majorities of the alveoli (A1) appeared normal with normal and thin inter-alveolar septum (arrow). Few alveoli (A2) suffered from degenerative changes and thickening of the alveolar wall (arrow-head). The pulmonary blood vessels showed a minimal degree of congestion (V). (**f**)—inhaled NFD nanocomposites + bleomycin group showed normal pulmonary tissues with normal bronchus and bronchiole (B). The lung alveoli (A) appeared normal with normal and thin alveolar epithelium and inter-alveolar septum (arrow). The alveoli containing alveolar macrophages (arrow-heads). (**g**–**l**): lung tissues stained with Masson’s trichrome. Magnification ×100. (**g**)—normal control group showed normal pulmonary tissues with fine collagenic fibers around the bronchioles and blood vessels (arrow). The inter-alveolar septum (arrow head) around alveoli (A) appeared free from any collagen fibers. (**h**)—Oral NFD suspension-treated group showed normal pulmonary tissues with fine collagenic fibers around the bronchioles and blood vessels (arrow). The inter-alveolar septum (arrow head) around alveoli (A) appeared free from any collagen fibers. (**i**)—bleomycin group showed proliferative collagenic bundles around bronchioles (B) and blood vessels (V) (arrow-head). The inter-alveolar septum around the alveoli (A) contained thick collagen bundles (arrow). (**j**)—oral NFD suspension + bleomycin group showed proliferative collagenic bundles around bronchioles (B) and blood vessels (V) (arrow). The inter-alveolar septum around few alveoli (A) contained thick collagen bundles (arrow head) while the others appeared normal. (**k**)—inhaled NFD suspension + bleomycin group showed proliferative collagenic bundles around bronchioles (B) and blood vessels (V) (arrow). The inter-alveolar septum around few alveoli (A) contained fine collagen fibers (arrow-head) while the majority of alveoli appeared normal. (**l**)—inhaled NFD nanocomposites + bleomycin group showed normal pulmonary tissues with fine collagenic fibers around the bronchioles and blood vessels (arrow). The inter-alveolar septum (arrow-head) around alveoli (A) appeared free from any collagen fibers. ONFDS: oral nifedipine suspension; BL: bleomycin; INNFDS: inhaled nifedipine suspension; INNFDNCs: inhaled nifedipine nanocomposites.

Moreover, Boren et al. [83] showed that the treatment of human lung fibroblasts with SB216763 impeded GSK-3β and mitigated pulmonary fibrosis, which was associated with the attenuation of α-SMA. Furthermore, GSK-3β was reported to modulate β-catenin [82]. Consistent with the above-mentioned studies, NFD nanocomposites in the existing study remarkably resulted in the suppression of GSK-3β, even though such modulation manifested to be statistically insignificant, Figure 6. The current biochemical investigations were supported by the histopathological findings and scoring, as NFD nanocomposites successfully restored the normal architecture of the lung to a greater extent than bleomycin, as well as oral and inhaled NFD suspensions, Figure 7. 

In the current work, the cytological findings of BAL revealed that the macrophages were the predominant cells in normal rats’ lavage, with a ratio of more than 90%, while the changes in other cells appeared insignificant. Those with increased percentages of neutrophils <20%, lymphocytes <30%, and desquamated epithelial cells <25% on expense to decreased macrophages of percentages >25% in the lungs suffered from non-specific fibrotic diseases, which appeared in rats treated with bleomycin (drug-induced fibrosis). Administrations of either oral NFD suspension, inhaled NFD suspension, or NFD nanocomposites ameliorated the action of bleomycin on lung tissues in which the percentages of macrophages, neutrophils and lymphocytes returned to the normal range, with a superior effect to that of the NFD nanocomposites, Table 8.

Collectively, our experimental work proved a plausible biological mechanism for the promising potential therapeutic effect associated with the use of intratracheal NFD nanocomposites in IPF.

## 4. Conclusions

In the current work, CTS-PLGA nanocomposites were successfully tailored and optimized for pulmonary targeting of NFD *via* a modified spontaneous solvent emulsification–evaporation technique. The optimized nancomposites formulation, constituted of 0.52% *w/v* PLGA and 1.5% *w/v* PVA, besides 0.25% *w/v* CTS, revealed a spherical morphology with an adequately high *EE%*, a small particulate size, and a prolonged release profile over 24 h. The nanocomposites divulged aerodynamic features as well as size distributions appropriate for pulmonary targeting. In vivo pharmacokinetic studies manifested overtly snowballed bioavailability of the optimal intratracheal nanocomposites of approximately 3.68-fold and extended T_1/2_ to 6.66 ± 0.98 h when compared to oral NFD suspension. In rat model, NFD secured against pulmonary fibrosis induced by bleomycin when simultaneously-administered with it in nanocomposites form, which had augmented potency. The molecular mechanism for this protection was through inhibition of TGF-β/β-catenin and oxidative stress signaling pathways. Hence, the designated CTS-PLGA nanocomposites could donate a tolerable and fruitful nanoplatform for lung delivery of NFD which can surpass its concomitant oral shortcomings, meanwhile fulfilling clinical benefits for pulmonary fibrosis management. 

## Figures and Tables

**Table 1:** The statistical design (3^2^ full factorial) parameters elected for optimization of NFD-CTS-PLGA nanocomposites.

Variable	Design Level		
	Low (−1)	Medium (0)	High (+1)
Independent variables			
*X*_1_: PLGA concentration (% *w*/*v*)	0.25	0.50	1.00
*X*_2_: PVA concentration (% *w*/*v*)	0.50	1.00	1.50
Dependent variables	Constraints		
*Y*_1_: *EE%*	Maximize		
*Y*_2_: Particle size (nm)	Minimize		
*Y*_3_: *Q*_24 *h*_ (%)	Maximize		

NFD: nifedipine; CTS: chitosan; PLGA: poly(D,L-lactide-co-glycolide); PVA: polyvinyl alcohol; *EE%*: entrapment efficiency percent; *Q*_24 *h*_: accumulative % release over 24 h.

**Table 2:** The observed responses of NFD-CTS-PLGA nanocomposites as per the implemented 3^2^ full factorial design.

Formulation	Independent Variables		Dependent Variables			PDI
	*X*_1_: PLGA Concentration (% *w*/*v*)	*X*_2_: PVA Concentration (% *w*/*v*)	*Y*_1_: *EE%*	*Y*_2_: Particle Size (nm)	*Y*_3_: *Q*_24 *h*_ (%)	
F1	0.25	0.50	62.97 ± 3.15	222.81 ± 12.01	50.43 ± 2.26	0.271
F2	0.25	1.00	57.58 ± 3.03	206.23 ± 11.46	58.73 ± 3.97	0.460
F3	0.25	1.50	53.62 ± 2.54	188.20 ± 10.34	63.65 ± 2.72	0.258
F4	0.50	0.50	72.04 ± 3.67	240.34 ± 9.88	40.21 ± 1.31	0.407
F5	0.50	1.00	66.31 ± 2.12	233.86 ± 17.58	45.22 ± 1.53	0.264
F6	0.50	1.50	61.29 ± 3.50	224.72 ± 14.06	53.74 ± 3.14	0.473
F7	1.00	0.50	77.18 ± 2.75	280.41 ± 11.63	30.50 ± 2.37	0.343
F8	1.00	1.00	71.90 ± 4.04	258.85 ± 19.14	39.32 ± 1.00	0.235
F9	1.00	1.50	68.13 ± 2.01	235.17 ± 13.67	41.45 ± 4.05	0.309

NFD: nifedipine; CTS: chitosan; PLGA: poly(D,L-lactide-co-glycolide); PVA: polyvinyl alcohol; *EE%*: entrapment efficiency percent; *Q*_24 *h*_: accumulative % release over 24 h; PDI: polydispersity index. Data are mean values (*n* = 3) ± SD.

**Table 3:** Regression analysis results for the response variables.

Model	Adequate Precision	R^2^	Adjusted R^2^	Predicted R^2^	SD	% CV	*p* Value	Remarks
Response (*Y*_1_)								
Linear	29.64	0.9054	0.8975	0.8852	2.37	3.60	<0.0001	-
2FI	25.13	0.9058	0.8935	0.8804	2.42	3.67	<0.0001	-
Quadratic	51.46	0.9852	0.9817	0.9756	1.00	1.52	<0.0001	Suggested
Response (*Y*_2_)								
Linear	34.09	0.9289	0.9229	0.9101	7.23	3.11	<0.0001	-
2FI	31.68	0.9395	0.9316	0.9196	6.80	2.93	<0.0001	-
Quadratic	36.64	0.9711	0.9642	0.9530	4.93	2.12	<0.0001	Suggested
Response (*Y*_3_)								
Linear	36.34	0.9367	0.9314	0.9241	2.65	5.64	<0.0001	-
2FI	31.13	0.9385	0.9305	0.9247	2.67	5.68	<0.0001	-
Quadratic	47.13	0.9827	0.9786	0.9722	1.48	3.15	<0.0001	Suggested

*Y*_1_: entrapment efficiency percent; *Y*_2_: particle size (nm); *Y*_3_: accumulative release over 24 h (%); R^2^: coefficient of determination; SD: standard deviation; CV: coefficient of variation.

**Table 4:** Composition, observed and predicted responses of the optimum NFD-CTS-PLGA nanocomposites.

Factor	Optimal Value	Response Variable	Observed Value	Predicted Value	% Prediction Error ^a^
*X*_1_: PLGA concentration (% *w*/*v*)	0.52	*EE%*	61.81	62.87	−1.71
*X*_2_: PVA concentration (% *w*/*v*)	1.50	Particle size (nm)	226.46	219.05	3.27
		*Q*_24 *h*_ (%)	50.40	51.73	−2.64

NFD: nifedipine; CTS: chitosan; PLGA: poly(D,L-lactide-co-glycolide); PVA: polyvinyl alcohol; *EE%*: entrapment efficiency percent; *Q*_24 *h*_: accumulative % release over 24 h. ^a^ Calculated as (Observed-Predicted/Observed) × 100.

**Table 5:** Aerodynamic characterization of the optimal NFD-CTS-PLGA nanocomposites.

Aerodynamic Character	Value
TED (µg)	1816.20 ± 230.33
TED as percentage of nominal dose (%)	90.81 ± 9.82
FPD (µg)	1461.68 ± 217.47
FPF (%)	80.48 ± 8.46
MMAD (µm)	1.12 ± 0.28

Data are mean values (*n* = 6) ± SD. TED: total emitted dose; FPD: fine particle dose; FPF: fine particle fraction; MMAD: mass median aerodynamic diameter.

**Table 6:** Mean pharmacokinetic parameters in rat plasma following administration of oral NFD suspension, intratracheal NFD suspension, and intratracheal NFD-CTS-PLGA nanocomposites.

Pharmacokinetic Parameter	Mean ± SD		
	Oral NFD Suspension	Intratracheal NFD Suspension	Intratracheal NFD-CTS-PLGA Nanocomposites
C_max_ (ng/mL)	1820.76 ± 240.52	3020.23 ± 590.47 ^a^	1198.22 ± 270.13 ^a,b^
t_max_ (h)	0.50 ± 0.00	0.50 ± 0.00	4.00 ± 0.00 ^a,b^
K_elim_ (h^−1^)	0.3414 ± 0.0434	0.2864 ± 0.0173	0.1041 ± 0.0121 ^a,b^
t_1/2_ (h)	2.03 ± 0.31	2.42 ± 0.14	6.66 ± 0.98 ^a,b^
AUC_0–24_ (ng h/mL)	2587.23 ± 261.67	3990.43 ± 441.25 ^a^	8203.52 ± 852.59 ^a,b^
AUC_0–∞_ (ng h/mL)	2589.53 ± 178.83	4035.03 ± 630.82 ^a^	9535.33 ± 940.32 ^a,b^
MRT (h)	3.29 ± 0.95	3.74 ± 0.76	13.04 ± 2.24 ^a,b^
F_rel_ (%)	--	155.82	368.23 ^b^

NFD: nifedipine; CTS: chitosan; PLGA: poly(D,L-lactide-co-glycolide). Listed data are mean values (*n* = 6) ± SD. Using one-way ANOVA followed by Tukey post-hoc test. ^a^ *p* < 0.05 versus oral NFD suspension. ^b^ *p* < 0.05 versus intratracheal NFD suspension.

**Table 7:** Biochemical analyses in different studied groups.

Groups	Hydroxyproline (μg/g Tissue)	MMP-7 (pg/g Tissue)	MDA (ng/g Tissue)	SOD (U/g Tissue)
Normal control	32.91 ± 7.10	57.24 ± 15.62	14.71 ± 3.19	31.15 ± 5.13
ONFDS	43.17 ± 9.24	60.33 ± 6.92	13.07 ± 1.57	29.50 ± 5.79
BL	183.99 ± 16.13 ^a,b^	685.93 ± 148.89 ^a,b^	50.21 ± 11.84 ^a,b^	7.46 ± 2.27 ^a,b^
BL + ONFDS	96.97 ± 18.03 ^a,b,c^	366.85 ± 131.66 ^a,b,c^	37.66 ± 8.87 ^a,b^	12.95 ± 3.67 ^a,b^
BL + INNFDS	66.99 ± 15.66 ^a,c,d^	206.62 ± 36.12 ^c,d^	31.37 ± 13.15 ^a,b,c^	16.50 ± 3.95 ^a,b,c^
BL + INNFDNCs	46.59 ± 12.17 ^c,d^	176.05 ± 75.33 ^c,d^	15.49 ± 6.50 ^c,d,e^	25.78 ± 5.54 ^c,d,e^

MMP-7: matrix metalloproteinase 7; MDA: malondialdehyde; SOD: superoxide dismutase; ONFDS: oral nifedipine suspension; BL: bleomycin; INNFDS: inhaled nifedipine suspension; INNFDNCs: inhaled nifedipine nanocomposites. ^a^ *p* < 0.05 versus normal control. ^b^ *p* < 0.05 versus ONFDS. ^c^ *p* < 0.05 versus BL. ^d^ *p* < 0.05 versus BL + ONFDS. ^e^ *p* < 0.05 versus BLM + INNFDS.

**Table 8:** The findings of bronchoalveolar lavage, degree of fibrosis (Ashcroft score) and histopathological scoring.

Group	Findings of Bronchoalveolar Lavage				Degree of Fibrosis (Ashcroft Score)	^a^ Histopathological Scoring			
	Desquamated Cells	Lymphocytes	Macrophages	Neutrophils		Bronchitis/Bronchiolitis	Edema	Epithelial Thickening	Epithelial Degeneration
Normal control	>5%	>3%	>90%	>3%	0	0	0	0	0
ONFDS	>10%	>3%	>85%	>3%	0	0	0	0	0
BL	<25%	<30%	>25%	<20%	4	3	3	3	3
BL + ONFDS	>15%	>10%	>70%	>5%	3	2	2	2	2
BL + INNFDS	>10%	>10%	>75%	>5%	3	1	2	2	2
BL + INNFDNCs	>5%	>5%	>85%	>5%	1	0	1	1	0

ONFDS: oral nifedipine suspension; BL: bleomycin; INNFDS: inhaled nifedipine suspension; INNFDNCs: inhaled nifedipine nanocomposites. ^a^ Histopathological scoring of lung tissue injury was scaled in degrees as follows: 0 = no change; 1 ≤ 25% tissue damage; 2 = 26–50% tissue damage; 3 = 51–75% tissue damage; 4 = 76–100% tissue damage.

## Data Availability

All processed data in this work are incorporated into the article.

## Supplementary Materials

The following is available online at https://www.mdpi.com/article/10.3390/ph14121225/s1, Figure S1: Particle size distribution curves of NFD-CTS-PLGA nanocomposites.
