# Supplementary figures and images for: Intratracheally Inhalable Nifedipine-Loaded Chitosan-PLGA Nanocomposites as a Promising Nanoplatform for Lung Targeting: Snowballed Protection via Regulation of TGF-β/β-Catenin Pathway in Bleomycin-Induced Pulmonary Fibrosis
Source: Pharmaceuticals (Basel). 2021 Nov 26;14(12):1225. doi: 10.3390/ph14121225 (PMC8707652; doi:10.3390/ph14121225)

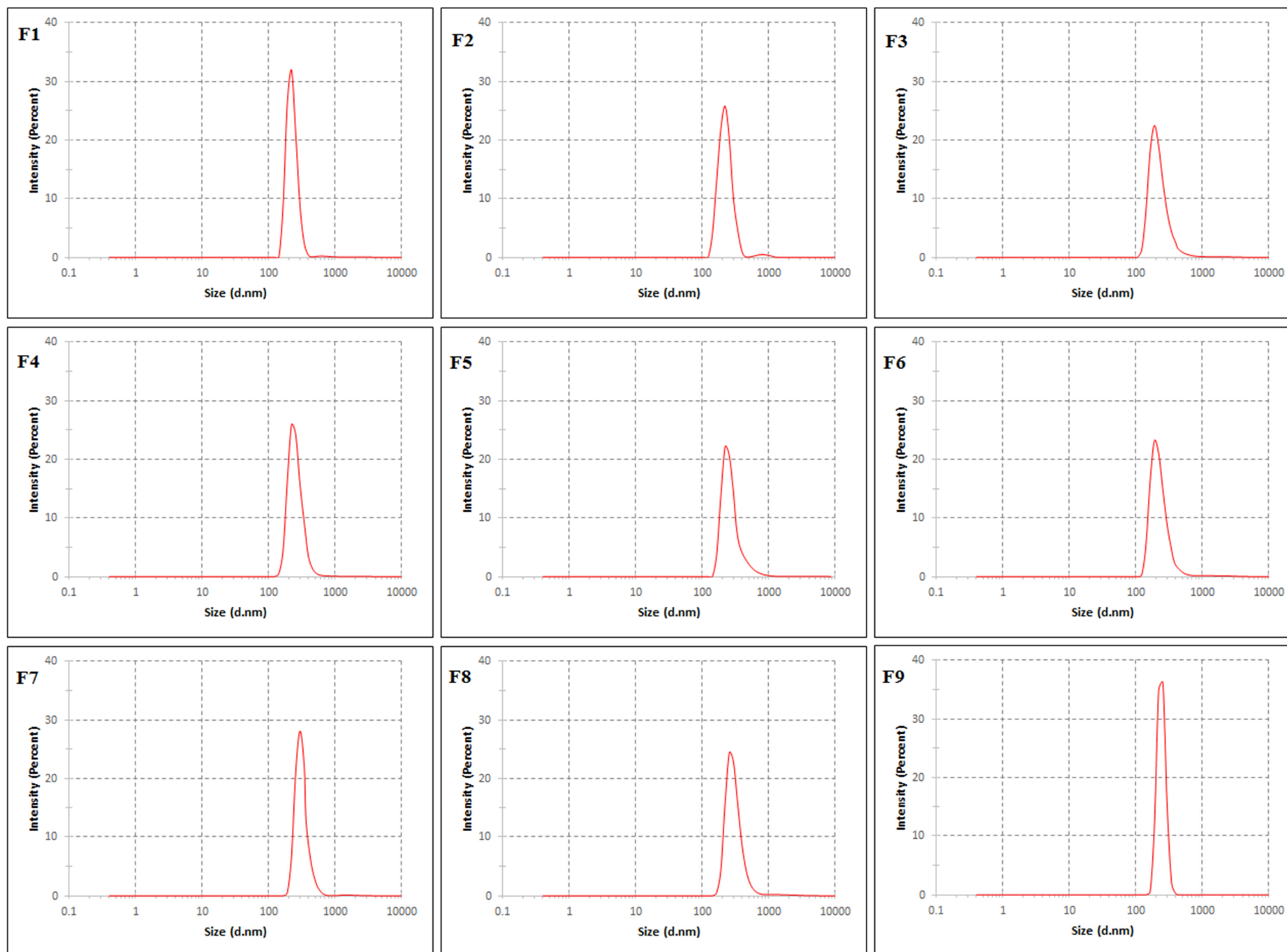

**Figure (S1):** Particle size distribution curves of NFD-CTS-PLGA nanocomposites.

Supplement: Supplementary file 1 [file pharmaceuticals-14-01225-s001.zip › pharmaceuticals-1445206-supplementary.pdf]
